# Creating heralded hyper-entangled photons using Rydberg atoms

**DOI:** 10.1038/s41377-021-00537-2

**Published:** 2021-05-12

**Authors:** Sutapa Ghosh, Nicholas Rivera, Gadi Eisenstein, Ido Kaminer

**Affiliations:** 1 Andrew and Erna Viterby Department of Electrical Engineering and Russell Berrie Nanotechnology Institute, Technion-Israel Institute of Technology, Haifa, 32000 Israel; 2 Department of Physics, Massachusetts Institute of Technology, Cambridge, MA USA

**Keywords:** Atom optics, Quantum optics

## Abstract

Entangled photon pairs are a fundamental component for testing the foundations of quantum mechanics, and for modern quantum technologies such as teleportation and secured communication. Current state-of-the-art sources are based on nonlinear processes that are limited in their efficiency and wavelength tunability. This motivates the exploration of physical mechanisms for entangled photon generation, with a special interest in mechanisms that can be heralded, preferably at telecommunications wavelengths. Here we present a mechanism for the generation of heralded entangled photons from Rydberg atom cavity quantum electrodynamics (cavity QED). We propose a scheme to demonstrate the mechanism and quantify its expected performance. The heralding of the process enables non-destructive detection of the photon pairs. The entangled photons are produced by exciting a rubidium atom to a Rydberg state, from where the atom decays via two-photon emission (TPE). A Rydberg blockade helps to excite a single Rydberg excitation while the input light field is more efficiently collectively absorbed by all the atoms. The TPE rate is significantly enhanced by a designed photonic cavity, whose many resonances also translate into high-dimensional entanglement. The resulting high-dimensionally entangled photons are entangled in more than one degree of freedom: in all of their spectral components, in addition to the polarization—forming a hyper-entangled state, which is particularly interesting in high information capacity quantum communication. We characterize the photon comb states by analyzing the Hong-Ou-Mandel interference and propose proof-of-concept experiments.

## Introduction

Entanglement is a unique feature of quantum mechanics that enables new possibilities in the fields of quantum information and quantum optics. In recent years, entangled multi-particle cluster states were used in quantum computation^1–3^. In other areas of quantum optics, entangled photons have been used to demonstrate quantum teleportation over a long distance^4–6^. Quantum hyper-dense coding protocols enable breaking the classical limit for information transfer and sharing more than one bit of information on a single qubit^7^. All these applications require efficient entangled photon sources, which are especially desirable in the telecommunications wavelengths, where photon propagation losses are low.

The need to create entangled photons motivated the study of quantum optics in different physical systems, such as hot vapor^8,9^, cold atoms^10^, semiconductors^11,12^, quantum dots^13^, nitrogen-vacancy centers in diamond^14^ and more^15–18^. In particular, the spontaneous four-wave mixing process has promising prospects since it is accessible in integrated platforms^15,16^. Currently, such schemes are promising for compact footprint relative to other technologies, but at the cost of lower emission rates. The most common approach to generate entangled photons is via spontaneous parametric down-conversion (SPDC) in nonlinear χ^(2)^ crystals^17^. In SPDC, pump photons interact with the quantum vacuum field inside a medium and down convert into photon pairs. The photon sources based on nonlinear process has several limitations. For example, even though SPDC sources are considered better in efficiency and emission rate relative to other mechanisms, they are still limited by the low nonlinear coefficient (∼10^−5^ pairs per one pump photon)^19,20^. Moreover, the SPDC process has a broad emission spectrum, which decreases the coherence length of the emitted photons and limits their usefulness for long-distance quantum communication^21^. This problem can be solved either by spectral filtering using optical cavities^22^ or by using very narrow bandpass filters, but typically at the price of reducing the number of photon pairs generated (thus further reducing the overall efficiency^15^).

The desired quality of quantum light sources is heralding, to confirm the generation of the quantum state without measuring it. Conventional entangled photon sources are not heralded. In the case of SPDC, heralding has been achieved by generating a trigger photon, but this process reduces the efficiency further^23,24^. Some heralding methods include the generation of multiple photon pairs, some of which are used for detection^25^.

An additional practical limitation of conventional mechanisms for the generation of quantum light is the output wavelength. The wavelength of the generated entangled photons depends on the choice of nonlinear optical crystals and by their phase-matching conditions. All these challenges create a need to explore new methods to generate more flexible quantum entangled sources of light, with the desire to achieve high efficiency, heralded sources, at telecommunications wavelengths.

Here we propose a new approach for creating a deterministic, heralded entangled photon source based on cavity-controlled two-photon spontaneous emission (TPE) by Rydberg atoms. In particular, by judicious design of the cavity and choice of the Rydberg atom, the TPE rates can be made higher than the competing one-photon rates. Moreover, the emitted entangled photon is at telecommunications wavelengths. Another cavity-enhanced transition is used for heralding to allow non-destructive detection of the entangled photons. Due to the multiple cavity resonances, each photon in the entangled pair forms a comb^26,27^, that is simultaneously entangled in energy and polarization degrees of freedom, thus forming hyper-entangled combs (simultaneous entanglement in many degrees of freedom). Although TPE is typically weak, especially compared to one-photon emission channels, we remedy this by means of a cavity that enhances TPE while it inhibits the competing one-photon processes and simultaneously enhances a consecutive emission in the near-IR, the latter of which helps in heralding.

The cavity QED framework we developed for the quantitative predictions is shared online^28^, and can be used for calculations of second-order QED processes with alkali atoms in any atomic state.

Hyper-entanglement is of great interest in light of current work^29–31^. Most of the quantum communication and quantum teleportation experiments use the entanglement in the polarization degree of freedom. However, it has been demonstrated that hyper-entanglement can increase the channel capacity beyond the limits of conventional entanglement^32–34^. To date, high-dimensional hyper-entangled photon pairs have been generated from nonlinear processes by entangling simultaneously the polarization and additional degrees of freedom such as multiple frequency modes of each photon^35^, spatial modes^36^, orbital angular momentum (OAM)^32^ and so on. In some works, the high-dimensional energy-entangled photons were produced by creating a photonic frequency comb^37,38^. These hyper-entangled states have been distributed over a long distance^39,40^ and also have been applied to quantum teleportation experiments^41,42^. Beyond work generating such hyper-entangled pairs, there are also protocols for purifying their entanglement, which helps to deterministically obtain maximally entangled pure states^43^. All these technological advances promote hyper-entanglement as a path towards robust quantum communication with higher channel capacity.

## Results

### The proposed design of the source of heralded hyper-entangled photons

**Fig. 1:**
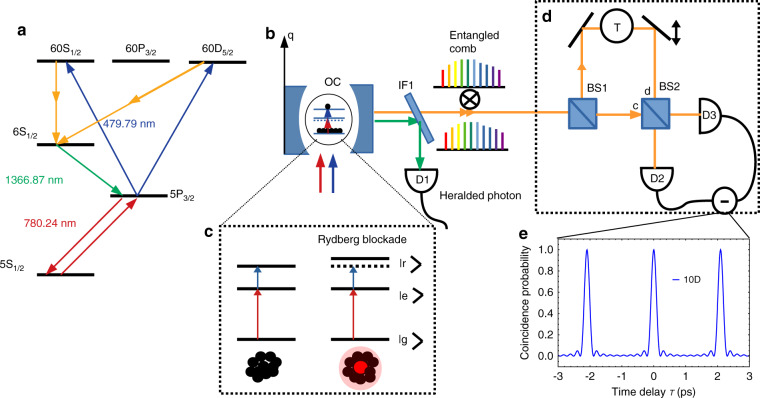
Schematics for the generation of heralded hyper-entanglement photon pairs. Schematics for the generation of heralded hyper-entanglement photon pairs. **a** The atom is optically pumped into a Rydberg state with probe beam (red) and the control laser (blue). From the 60S_1/2_ (60D_5/2_) state, due to an optical cavity, the atom decays to 5P_3/2_ by emitting an entangled photon pair (yellow) and a heralding photon (green). **b** The proposed experimental setup where atoms are trapped inside an optical cavity (OC) which is made resonant with the two-photon transitions from 60S_1/2_→6S_1/2_ and the heralding photon. All the dominant first-order transitions are inhibited as they are off-resonant with the cavity resonances. IF: Interference filter, D: Single-photon counting module (SPCM), BS: Non-polarizing beam splitter. **c** A scheme describing the Rydberg blockade. The excitation of even just a single Rydberg atom shifts the energy states of the neighboring atoms, inhibiting further Rydberg excitation within a blockade radius. **d** Frequency entanglement is detected by measuring the coincidence probability as a function of delay τ, between the paths of the two entangled photon combs. **e** The coincidence probability for a ten-dimensional entangled photon state.

The proposed method for the high-dimensional entangled photon generation is shown in Fig. 1. It starts with preparing atoms in the excited Rydberg state 60S_1/2_ (can also achieve a similar scheme with 60D_5/2_) of rubidium atoms with two input fields, a probe beam at wavelength 780 nm and a control field at 479.79 nm. These laser beams are collectively absorbed by all the atoms with an absorption probability enhanced by *N*_*a*_ (where *N*_*a*_ is the total number of atoms). Only one atom is excited to a Rydberg state due to the phenomenon of Rydberg blockade^44^. The *N*_*a*_-atom ground state, $\left| G \right\rangle = \left| {g_1,g_2, \ldots ,g_{N_a}} \right\rangle$ couples to the many-body collective excited state $\left| e \right\rangle = \frac{1}{{\sqrt {N_a} }}\mathop {\sum}\nolimits_{j = 1}^{N_a} {{\rm{e}}^{{\rm{i}}k.x_j}} \left| j \right\rangle$ where $\left| j \right\rangle = \left| {g_1,g_2, \ldots ,r_j, \ldots ,g_{N_a}} \right\rangle$ is the state with the *j*th atom in the Rydberg state, $r_j$. We denote *k* as the sum of the wave vectors of the probe and control fields and *x*_*j*_ as the position of the *j*th atom. Rydberg excitation of one atom shifts the energy level of the nearby atoms due to the dipole–dipole interaction. The shift in the Rydberg state of the neighboring atoms is large and proportional to $1/R^3$, where *R* is the distance between the atoms with the Rydberg atom. This inhibits other Rydberg excitations in the atomic cloud within a blockade radius^45^. The dipole–dipole interaction between the Rydberg atoms has been explored to implement fast quantum gates in neutral atom^46^ and also to perform quantum information processing based on collective excitations in mesoscopic atomic ensembles^47–49^ in the blockade region and also in the anti-blockade region^50–53^.

The lifetime of the 60S_1/2_ state is 100 µs (with a background temperature of 300 K), after which the atom decays back to the ground state by multiple one-photon emission processes. But if the atom is prepared inside an optical cavity with high finesse, such that the sum of two cavity frequency modes is equal to the transition frequency between the state 60S_1/2_ and 6S_1/2_, then the atom decay will be dominated by TPE instead of one-photon emission. The emitted photon state is in general a superposition of all the possible frequency pairs supported by the cavity. This generates a high-dimensional entangled photon where the dimensionality of the photon state is given by the number of cavity resonances.

From 6S_1/2_, the atom further decays to 5P_3/2_ with the emission of light at 1366.87 nm. We design the cavity to also be resonant at this wavelength so that the emission rate is enhanced by the Purcell effect. Consequently, this transition dominates over all other decay pathways from 6S_1/2_, and the 1366.87 nm photon is practically emitted instantaneously after the entangled photon pair (relative to all other time scales in the problem) and confirms the generation of an entangled pair without directly measuring it. In practice, the lifetime of this transition determines the heralding time resolution, i.e., the temporal precision by which the detection of the heralding photon predicts the existence of the entangled pair. From this point, the atom at the 5P_3/2_ state is again collectively excited to the Rydberg state and the whole process is repeated.

One can prepare a cloud of cold Rb-87 atoms in a magneto-optical trap which is formed at the center of the cavity to achieve a maximum coupling with the cavity fields. The laser beams at 780.24 nm and 479.79 nm excite an atomic ensemble along the plane perpendicular to the cavity axis. Photons from both beams are collectively absorbed by the ensemble of atoms. The interaction of atoms with light can be stabilized by trapping atoms in the dipole trap formed by a far red-detuned light along the three dimensions. The dipole trap forms a conservative potential that does not interact with the light, trapping the atom into a high-intensity region. Since the size of the Rydberg atoms is around a few µm, there is no need to confine the atoms in the trap beyond this length scale. Altogether, the Rydberg blockade phenomenon allows single Rydberg excitation, from which the decay is controlled by the cavity spectrum. Both the heralding photon and the entangled pair are emitted along the cavity axis, and can be separated by a color filter.

### Theory of the TPE rates from Rydberg atoms in cavities

Multi-photon spontaneous emission processes for atom-based systems are generally very slow. This is due to the mismatch of atom size *a* and the wavelength of the light λ. The probability of spontaneous decay via *n* photons goes as $\alpha^n(a/\lambda )^{2n}$, where *α* is the fine structure constant and is equal to 1/137^54^. Since the ratio $a/\lambda = 1/1000$, the probability for two-photon emission (TPE) is smaller by a factor 10^8^ than the one-photon processes. But the ratio $a/\lambda$ can be increased either by decreasing the wavelength of light as in the case of polaritons^55^ or by exciting the Rydberg state of atoms that have large atomic sizes (∼µm). The Rydberg states are formed by exciting the atoms to higher energy levels, where the lifetime of the excited states increases (∼ms). Due to the larger effective atom size, the dipole moment of the atoms also increases, which in turn enhances the atom–light interaction^56^.

The TPE processes in atoms were studied rigorously in the literature^57–59^. The atoms from the excited state decays to a ground state either through a resonant cascade process where the intermediate state lies between the initial and final energy state, or by passing through an intermediate state having higher energy than the initial state. The latter is called non-resonant TPE. Although the non-resonant processes are very slow compared to the resonant ones, they also contribute to the lifetime of the excited state. To calculate the transition rates, we use second-order quantum electrodynamics (QED) with enhancements by a photonic cavity, quantitatively described by the theory of macroscopic QED^60^, which in our case also conforms with cavity QED.

The Hamiltonian describing the atom–light composite system is $H_{{\rm{total}}} = H_{{\rm{atom}}} + H_{{\rm{light}}} + H_{{\rm{int}}}$, where $H_{{\rm{atom}}} = \frac{{p^2}}{{2m_{\rm{e}}}} + V(r)$ is the atomic Hamiltonian and *m*_e_ is the electron mass. The exact form of $V(r)$ for Rydberg state of Rb atom is given in supplementary III. $H_{{\rm{field}}} = \mathop {\sum}\nolimits_{k,\sigma } {\hbar \omega _k(a_{k,\sigma }^\dagger a_{k,\sigma } + \frac{1}{2})}$ is the Hamiltonian corresponding to an electromagnetic field with wave vector *k*, frequency $\omega _k$ and polarization $\sigma$. The atom–light interaction is described as $H_{{\rm{int}}} = \mathop {\sum}\nolimits_i {\frac{e}{{2m_{\rm{e}}}}(p_i.A\left( {r_i} \right) + A\left( {r_i} \right).p_i)}$, where *e* is the electronic charge and the vector potential $A\left( {r_i} \right) = \mathop {\sum}\nolimits_{k,\sigma } {\sqrt {\frac{\hbar }{{2{\it{\epsilon }}_0\omega _kV}}} (a_{k,\sigma }u_{k,\sigma }{\rm{e}}^{ - {\rm{i}}k.r} + {\rm{h.c.}})}$ represents all the vacuum modes around the atom in volume *V*.

The TPE rates from the initial state $\left| i \right\rangle$ to the final state $\left| f \right\rangle$, with an energy gap $\omega _{if}$, can be calculated using second-order perturbation theory in QED (elaborated in Supplementary section I):

1 $$\begin{array}{l}\frac{{{\rm{d}}{\Gamma} }}{{{\rm{d}}\omega _1}} = \frac{{2\pi }}{{\hbar{\,}^4}}\;\left| \mathop {\sum}\limits_m \frac{{ < f|H_{{\rm{int}}}^{\left( 2 \right)}|m > < m|H_{{\rm{int}}}^{\left( 1 \right)}|i > }}{{\omega _1 + \omega _{mi}}}\right.\\\qquad\quad \left.+ \frac{{ < f|H_{{\rm{int}}}^{\left( 1 \right)}|m > < m|H_{{\rm{int}}}^{\left( 2 \right)}|i > }}{{\omega _{if} - \omega _1 + \omega _{mi}}} \right|^2\rho \left( {\omega _1} \right)\rho (\omega _{if} - \omega _1)\end{array}$$

$\rho \left( \omega \right)$ represents the density of photonic states. By modifying $\rho \left( \omega \right)$ with an appropriate cavity, one can engineer the spontaneous emission rate of the atom. The above equation includes the contribution from all the intermediate virtual states, $m \ge i$ and the resonant states, $m \,<\, i$ for the two-photon processes. For states, $m \,<\, i$, the intermediate resonant states are added as a small imaginary contribution to their energy in the denominator^58^.

For an atom in free space, the TPE rate is obtained from Eq. (1) as

2 $$\begin{array}{l}{\Gamma} _{{\rm{free}}} =\frac{{3^2Z^{10}}}{{2^{11}}}a_lR_H\alpha ^6c\left( {\frac{{\omega_{if}}}{{\omega _0}}} \right)^5\mathop {\int}\limits_{\omega =0}^{\omega _{if}} \omega ^3\left( {\omega _{if} - \omega } \right)^3\\\qquad{\rm{d}}\omega \left| \mathop {\sum}\limits_m {d_{fm}d_{mi}}\left( {\frac{1}{{\omega - \omega _{im}}} + \frac{1}{{\omega _{if} -\omega - \omega _{im}}}} \right) \right|^2\end{array}$$

where, $a_l$ is the contribution from the average angular part of the wave function which is 1 for a final state with orbital quantum number *l* = 0 and 2/5 for *l* = 2. The term $d_{fm} = f\left| r \right|m$ represents the dipole matrix element for transition from the virtual state *m* to the final state *f*. The detailed calculation is given in Supplementary section II. For Rydberg states, the dipole matrix is calculated by solving the Schrodinger equation for *H*_atom_ using the Numerov method. We used the Python library from reference^61^ to calculate the dipole matrix for each state m. Details are given in Supplementary section III.

**Fig. 2:**
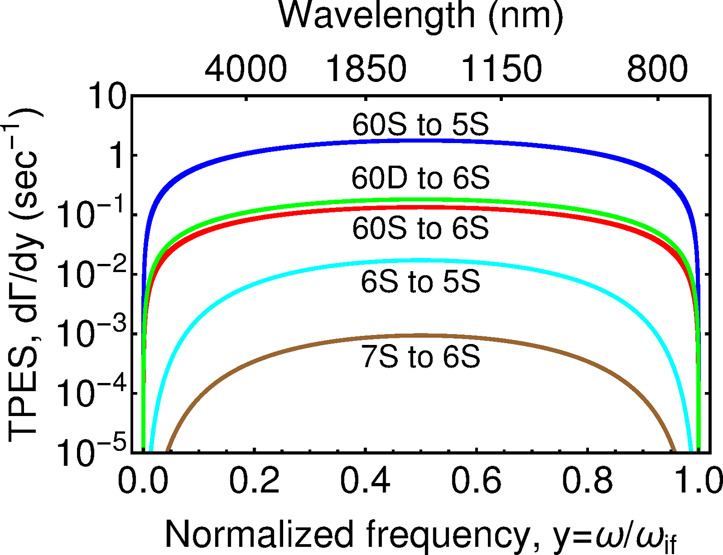
Comparison between the two-photon emission (TPE) spectra of Rb-87: emission from Rydberg states and from lower energy states. Comparison between the two-photon emission (TPE) spectra of Rb-87: emission from Rydberg states and from lower energy states. The TPE rates are higher for Rydberg state than for lower energy states by 2 orders of magnitude. The horizontal axis represents the dimensionless frequency *y* which is normalized by the frequency difference between the initial and final state $\omega _{if}$. The vertical axis is the rate of TPE per unit normalized frequency.

The TPE spectrum as a function of frequency was calculated from Eq. (2) and shown in Fig. 2. The total TPE rates obtained by integrating over the frequency are summarized in Table 1. The table shows that the one-photon emission rates from the Rydberg state to lower energy levels are less than the transitions from the low principle quantum number state. This decrease is due to the small overlap of the wave functions between the Rydberg states and the lower energy electron orbitals. In comparison with one-photon transition rates to lower energy states, the TPE rates are higher by two orders of magnitude^62^. This is because the TPE rates involve intermediate transitions between Rydberg-Rydberg states which have higher dipole moments as explained before. Nevertheless, when comparing to the one-photon transitions into relatively high energy levels (near the initial Rydberg states), the TPE rates are still smaller by a factor of 10^5^. Therefore, for an atom in free space, the dominant decay channel is still through the one-photon process.

**Table 1:** Two-photon emission (TPE) rates for a rubidium atom in free space.

Transition	Two-photon emission rate (s^−1^)	Transition	One-photon emission rate (s^−1^)
6S_1/2_ → 5S_1/2_	0.00454	6S_1/2_ → 5P_3/2_	7.4 × 10^6^
7S_1/2_ → 6S_1/2_	0.000241	7S_1/2_ → 5P_3/2_	6.4 × 10^6^
60S_1/2_ → 5S_1/2_	0.5902	60S_1/2_ → 5P_3/2_	1338.13
60S_1/2_ → 6S_1/2_	0.0447	60S_1/2_ → 6P_3/2_	517

TPE rates for transitions from the Rydberg states are higher than transitions from lower energy levels, however, the decay from the Rydberg states is still dominated by the one-photon processes.

For an atom inside an optical cavity, the density of the optical modes alters the spontaneous emission rates^63,64^. It is possible to engineer the cavity such that it enhances the two-photon processes and inhibits the one-photon processes. The enhancement of the TPE spectrum by the cavity scales like $\propto F(\omega _1)F(\omega _2)$ where $F\left( \omega \right) = \frac{{3Q\lambda ^3}}{{4\pi V}}$ is the Purcell factor for cavity mode $\omega$ at resonance. To exemplify the effect of the cavity, we consider the following parameters: cavity quality factor $Q = 10^8$, wavelength $\lambda = 1.55\;{\rm{\mu m}}$, mode volume $V = \pi \omega _{{\rm{cav}}}^2L/4$ with $\omega _{{\rm{cav}}} = 2{\rm{\mu m}}$ being the beam waist at the center of the cavity. Substituting these values into the Purcell factor formula yields an enhancement factor of $10^7$. The cavity-enhanced TPE rates are calculated from Eq. (1) (derivation shown in Supplementary section V):

3 $$\begin{array}{l}{\Gamma} _{{\rm{cav}}} = \frac{{3^4\pi ^2R_H\alpha ^6}}{{2^{10}}}\left( {\frac{{\omega _{if}}}{{\omega _0}}} \right)^5\left( {\frac{1}{{V\omega _{if}}}} \right)^2{\int}_{\omega = 0}^{\omega _{if}} {\omega \left( {\omega _{if} - \omega } \right){\rm{d}}\omega \;\mathop {\sum}\limits_{n_1} {\mathop {\sum}\limits_{n_2} {Q_{n1}Q_{n2}} } \left( {\frac{{\omega _{if}}}{{\omega _{cn1}}}} \right)\left( {\frac{{\omega _{if}}}{{\omega _{cn2}}}} \right)} \\ \frac{1}{{\left[ {1 + \left( {\frac{{\omega _{cn1}^2 - \omega ^2}}{{\omega \kappa }}} \right)^2} \right]\left[ {1 + \left( {\frac{{\omega _{cn2}^2 - (\omega _{if} - \omega )^2}}{{(\omega _{if} - \omega )\kappa }}} \right)^2} \right]}}\left| {\mathop {\sum}\limits_m {d_{fm}d_{mi}} \left( {\frac{1}{{\omega - \omega _{im}}} + \frac{1}{{\omega _{if} - \omega - \omega _{im}}}} \right)} \right|^2\end{array}$$

The cavity-enhanced total TPE rates have been calculated from Eq. (3), for different values of finesse and have been summarized in Table 2. It is evident that with proper cavity parameters, the TPE rates can be made significantly higher than the one-photon process. The cavity length has been chosen such that all the relevant first-order processes are made off-resonant. As a result, the emission rates for the first-order processes decrease by a factor *1/Q*^65^. This type of approach was already shown feasible when it was used to prolong the lifetime of Rydberg states by a factor of 20^66^. The cavity suppression of the first-order processes from 60S_1/2_ is summarized in Supplementary section VII (Table S2). The one-photon transitions that are not inhibited by the cavity have rates that are lower than the TPE rate and can be neglected.

**Table 2:** Comparison between the TPE rates and the one-photon rates for rubidium atom inside the cavity.

Finesse	TPE for 60S_1/2_ → 6S_1/2_ (s^−1^)		Inhibited trans., 60S_1/2_ → 5P_3/2_ (10^−6 ^s^−1^)	
	w/o cavity	With cavity	w/o cavity	With cavity
1.2 ×10^6^	0.0081024	123.25	1338.13	1.34
120 ×10^3^	0.0081024	12.32	1338.13	13.4
12 ×10^3^	0.0081024	1.23	1338.13	133.80

The cavity length *L* is tuned such that it enhances the TPE rates and the heralding photon while it inhibits the one-photon rates.

The TPE rate, ${\Gamma} _{{\rm{cav}}}$ depends on the product of the quality factors of the cavity for the two-photon modes. Hence, by increasing the reflectivity of the mirrors, the rates can be further enhanced. The rate is inversely proportional to the square of the mode volume, *V* which depends on the mode waist, defined as $\omega _{{\rm{cav}}} = \sqrt {\frac{\lambda }{{2\pi }}} \left( {L\left( {2R - L} \right)} \right)^{1/4}$. Small mode waists can be obtained by either using short cavities, or by choosing *L* close to the edge of the stability region$L \approx 2R$, where *R* is the radius of curvature of the cavity mirror^67,68^.

**Fig. 3:**
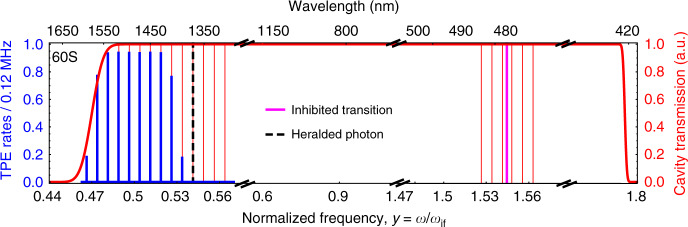
Enhancement of the two-photon emission rates (TPE) for a Rydberg atom (60S1/2 → 6S1/2) inside a cavity. Enhancement of the two-photon emission rates (TPE) for a Rydberg atom (60S_1/2_→6S_1/2_) inside a cavity. Cavity-enhanced TPE generates an entangled photon comb (blue); the TPE enhancement at each frequency depends both on the cavity Purcell factor at this frequency and on the Purcell factor at the frequency of the other photon in the pair. The cavity length is tuned such that the heralding transition at 1366.87 nm (black dashed) is enhanced by the cavity while all the other one-photon decay transitions are inhibited by being off-resonant. In particular, the fastest one-photon process 60S_1/2_→5P_3/2_ at 479.79 nm is also inhibited by the cavity (magenta). The envelope of the cavity transmission spectrum is shown in red.

Figure 3 shows that each emitted entangled photon is a superposition of all the possible modes of the cavity. This creates a *N*-dimensional entangled photon pair state where *N* is the number of cavity modes. The TPE spectrum is determined by the cavity spectrum multiplied with the TPE spectrum in the free space (shown in Fig. 2). The dimension of each entangled pair can be increased using cavities that have a broader spectrum, thus supporting more frequency modes. This will also increase the TPE rates. The choice of appropriate cavity spectrum also gives the flexibility to tune the wavelength of the emitted photons. If a different wavelength range is required, we can redesign the cavity by using other rubidium Rydberg energy levels. The cavity length is fixed such that the sum of the two cavity modes is equal to the energy difference, $\omega _{if}$. As an example, we have created a ten-dimensional entangled state with a cavity length 98.417 µm. The photon pair production rate for each energy mode is 120 kHz with a coherence time of 0.8 µs. The heralding photon emission rate is enhanced by a factor of 2 × 10^5^. The entangled photons emitted from the cavity can be characterized in detail by using the approach recently developed^69^. This opens an opportunity to perform quantum measurements with entangled photon comb pairs.

### Characterization of the Entanglement

The *N*-dimensional entangled state can be characterized with a Hong Ou Mandel (HOM) interferometer where the entangled photons are superimposed on a non-polarizing beam splitter (BS)^16^ and detected by detectors D2 and D3 as shown in Fig. 1d. A time delay, τ is introduced between the two photons. The interference spectrum as a function of delay between the two paths of the photon verifies the entanglement. The *N*-dimensional two-photon entangled state is written as

4 $$\left| \psi \right\rangle = \frac{1}{N}\mathop {\sum}\limits_{n = 1}^{N/2} {\alpha (\omega _n)} \left[ {a_c^\dagger \left( {\omega _n} \right)a_d^\dagger \left( {\omega _{if} - \omega _n} \right) + {\rm{e}}^{{\rm{i}}\phi \left( {\omega _n} \right)}a_d^\dagger \left( {\omega _n} \right)a_c^\dagger (\omega _{if} - \omega _n)} \right]\left| {0,0} \right\rangle$$

where *c* and *d* are the two input ports of the BS, $\alpha (\omega _n)$ is the amplitude coefficient for each pair of entangled photon mode which depends on the spectral properties of the cavity mirror, $\phi \left( {\omega _n} \right)$ represents the phase acquired by the photon due to the transition from different virtual states. For simplicity, we consider $\phi = 2\pi m$, where *m* is an integer. If all the energy modes are equally probable then the coincidence probability is calculated by averaging over the quantum states as shown in Supplementary section VI and is given by

5 $$P_2(\tau ) = \left\langle {E_d^ - (t)E_c^ - \left( {t + \tau } \right)E_c^ + \left( {t + \tau } \right)E_d^ + (t)} \right\rangle = \frac{1}{N}\left[ {1 + \frac{2}{N}\mathop {\sum}\limits_{\begin{array}{*{20}{c}} {i = j} \\ {i \ne j} \end{array}}^{N/2} {\mathop {\sum}\limits_{j = 1}^{N/2} {\cos \left( {\omega _i - \omega _j} \right)\tau } } } \right]$$

**Fig. 4:**
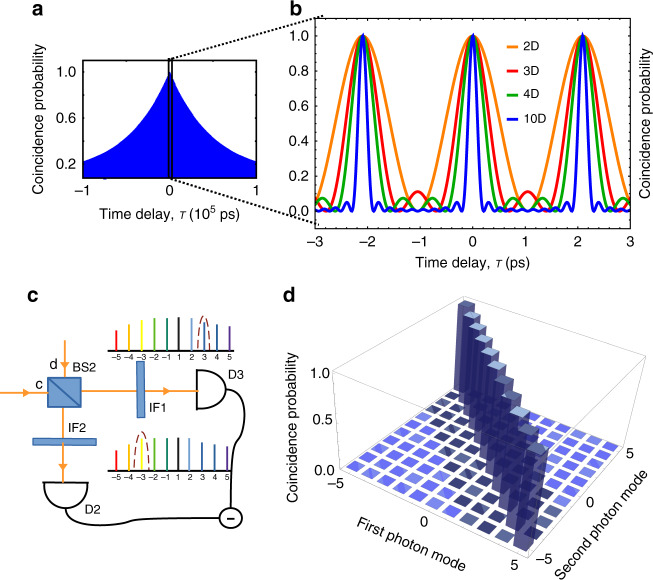
The coincidence probability P2 (τ) for an entangled comb. The coincidence probability P_2_ (τ) for an entangled comb. **a** plotted over a large time delay which shows a slow decrease in coherence due to the narrow width of cavity resonances. **b** Comparison of P_2_(τ) between two (orange), three (red), four (green) and ten (blue) dimensional entangled photon states. **c** Coincidence measurement setup (shown in Fig. 1) represents the mixing between different comb modes. **d** Coincidence measurement for the 10 entangled comb pairs which are generated from the cavity length 98.417 µm. The small off-diagonal components represent mixing coming from the bandwidth of the optical filter used for the measurement (chosen here to be 100 pm).

The frequency modes of the *N*-dimensional photon comb are separated by the cavity’s free spectral range. Thus, the coincidence probability for ten-dimensional entangled pair is $P_2\left( \tau \right) = \frac{1}{5}\left[ {1 + V_s\;f\left( \tau \right)\frac{1}{5}\mathop {\sum}\nolimits_{n = 1}^9 {\cos (2\pi n\nu _{{\mathrm{FSR}}}\;\tau )} } \right]$, where $f\left( \tau \right)$ represents the width of each energy mode. In our case, the energy filter is formed by the high finesse cavity, which is a narrow filter with Lorentzian shape. Therefore, $f(\tau ) = {\rm{e}}^{ - \kappa \left| \tau \right|}$ where κ is the cavity linewidth. The narrow cavity peaks give high coherence as shown in Fig. 4a. $V_s$ represents the visibility which is the probability of producing an entangled photon pair in each event. The visibility is directly related to the entanglement fidelity. Different noises can reduce the fidelity and each has a corresponding signature on the visibility. The visibility is also connected to the source efficiency, which depends on coupling losses and propagation losses. The coincidence probability for different photon pairs with different dimensions has been plotted in Fig. 4b. The correlation between different photon frequencies was calculated from the coincidence probability between photons filtered at the two frequencies as shown in Fig. 4c, d.

**Fig. 5:**
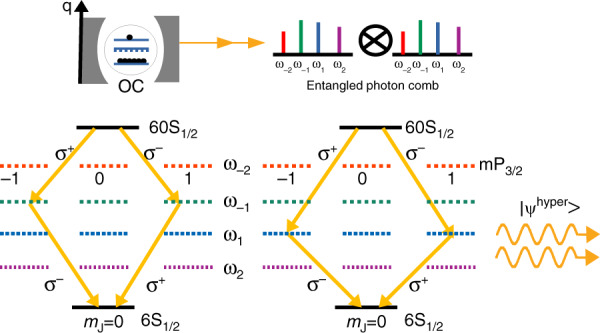
Generation of hyper-entangled state. Generation of hyper-entangled state. Polarization entanglement along with the *N*-dimensional energy-entangled state is created by setting the quantization axis *q* of the atom to be perpendicular to the cavity axis. This choice enables only σ± polarization excitations. The hyper-entangled state is given by Eq. (6).

Finally, we show that in addition to energy entanglement, the photons can also be entangled in polarization. When the atom’s quantization axis *q* is chosen to be perpendicular to the cavity axis, the atom at 60S_1/2_ state has pairs of choices that result in the same frequencies, yet with different Fig. 5. Each pair of emitted frequencies, $\omega _{ - 1}$ and $\omega _1,$ can be emitted in an alternating order and with opposite polarizations $\sigma _ +$ and $\sigma _ -$ ($| {1_{\omega_{-1},\sigma _ + },1_{\omega_1,\sigma _ - }} \rangle$ and $| {1_{\omega_{-1},\sigma _ - },1_{\omega_1,\sigma _ + }} \rangle$).

This creates polarization entangled states as the atom can decay via any two channels. The two-photon states represented by the two choices are given by $\left| {\psi _{{\rm{pol}}}^1} \right\rangle = \frac{1}{{\sqrt 2 }}\left( {\left| {\sigma _{\omega _{ - 1}}^ + \sigma _{\omega _1}^ - } \right\rangle \left| { \,+\, \left| {\sigma _{\omega _{ - 1}}^ - \sigma _{\omega _1}^ + } \right\rangle } \right.} \right)$ and $\left| {\psi _{{\rm{pol}}}^2} \right\rangle = \frac{1}{{\sqrt 2 }}\left( {\left| {\sigma _{\omega _1}^ + \sigma _{\omega _{ - 1}}^ - } \right\rangle \left| {\, +\, \left| {\sigma _{\omega _1}^ - \sigma _{\omega _{ - 1}}^ + } \right\rangle } \right.} \right)$ i.e., a simultaneous entanglement in energy and polarization known as hyper-entanglement. More generally, hyper-entangled state is defined as the entanglement in more than one degree of freedom. The hyper-entangled state for our entangled comb photons is written as

6 $$| {\psi ^{{\rm{hyper}}}} \rangle = \frac{1}{{\sqrt N }}\;\mathop {\sum}\limits_{n = 1}^{N/2} {\alpha _n\left( {| {\omega _n\;\omega _{ - n}} \rangle + | {\omega _{ - n}\omega _n} \rangle } \right)} \otimes \frac{1}{{\sqrt 2 }}\left( {| {\sigma ^ + \sigma ^ -} \rangle + | {\sigma ^ - \sigma ^ + } \rangle } \right)$$

In a similar way, hyper-entangled states can be prepared in 60D_5/2_ with *m*_*F*_ *=* 0. The hyper-entangled state has been used in quantum dense coding to measure all the Bell state pairs produced in one degree of freedom^32^. Using cross-Kerr nonlinearity, it is also possible to distinguish all the hyper-entangled Bell states in multiple degrees of freedom^70,71^. This enables us to perform quantum communication with larger channel capacity. Further research can explore the opportunities opened by heralded sources of hyper-entangled photon pairs.

## Discussion

The TPE rates are calculated above by using the dipole approximation. For Rydberg states, the size of the electron wave function can become quite large and even comparable to the wavelength, and therefore, it is interesting to extend the calculation beyond the dipole approximation in future work, as was done before in various cases^54^. Previous work^62^ showed that the continuum states play an important role in TPE rates from the hydrogen atom. Our calculations show that they do not play a comparable role in the case of the rubidium Rydberg state. The reason can be understood since for rubidium states the continuum wave functions oscillate much faster and its contribution averages out. This analysis is explained in more detail in Supplementary section IV. The efficiency of TPE processes can be reduced by other decay channels such as collision-induced decay and blackbody-induced decay. The collision with background gas is very low since the atoms are trapped in low vacuum pressure. The blackbody-induced decays are significant in our case. In the presence of blackbody radiation, the atom can absorb a photon and get excited to a higher Rydberg state. But, this can be suppressed by going to a cryogenic environment^72^, which increases the fidelity of the photon source at the cost of making it difficult to build into an integrated platform. The entanglement in different degrees of freedom (e.g., polarization and frequencies) are independent of each other, and thus we may model the decoherence of each degree of freedom separately. The multi-layer coating of the high reflective cavity mirrors can induce group velocity dispersion^73^. The dispersion can shift the various cavity modes differently depending on their frequency^74^, and can introduce a time delay between the entangled photons. The dispersion effects can be compensated by adding dispersive medium with an opposite sign of dispersion^75,76^ or by using mirror coatings with low dispersion.

In conclusion, we have proposed a new approach for the efficient generation of high-dimensional hyper- entangled photons at telecommunication wavelengths, which are heralded. The advantages of our approach arise because the TPE rates for Rydberg states are higher than for normal atomic states. The TPE is further enhanced by placing the atom inside an optical cavity that is tuned in a way that all the other dominant first order processes are inhibited. This makes the decay by TPE become the dominant process. The heralding of TPE allows non-destructive detection. We have also shown that this approach produces hyper-entangled states constructed from simultaneous polarization entanglement and energy entanglement. The combination of these advantages makes our proposed source promising in areas of quantum optics and quantum information.

## Materials and methods

The rubidium bound state wave functions are calculated numerically through the Numerov method. Various Python routines in the ARC package^61^ were used to calculate the wave functions, dipole matrix elements and emission rates.

Note – The formalism we developed is shared online as the Python package “TPE-Rydberg” that enables a range of quantitative predictions in Rydberg cavity QED^28^.

## Supplementary information

Supplementary information for Creating heralded hyper-entangled photons using Rydberg atoms
