# Correction to: Creating heralded hyper-entangled photons using Rydberg atoms

**DOI:** 10.1038/s41377-021-00569-8

**Published:** 2021-06-21

**Authors:** Sutapa Ghosh, Nicholas Rivera, Gadi Eisenstein, Ido Kaminer

**Affiliations:** 1 Andrew and Erna Viterby Department of Electrical Engineering and Russell Berrie Nanotechnology Institute, Technion-Israel Institute of Technology, Haifa, 32000 Israel; 2 Department of Physics, Massachusetts Institute of Technology, Cambridge, MA USA

**Keywords:** Atom optics, Quantum optics

Correction to: *Light: Science & Applications*

10.1038/s41377-021-00537-2 published online 12 May 2021

Following publication of this article^1^, it is noticed the Fig. 2 contained an error: three of the curves were cut. The correct Fig. 2 has been provided in this Correction.
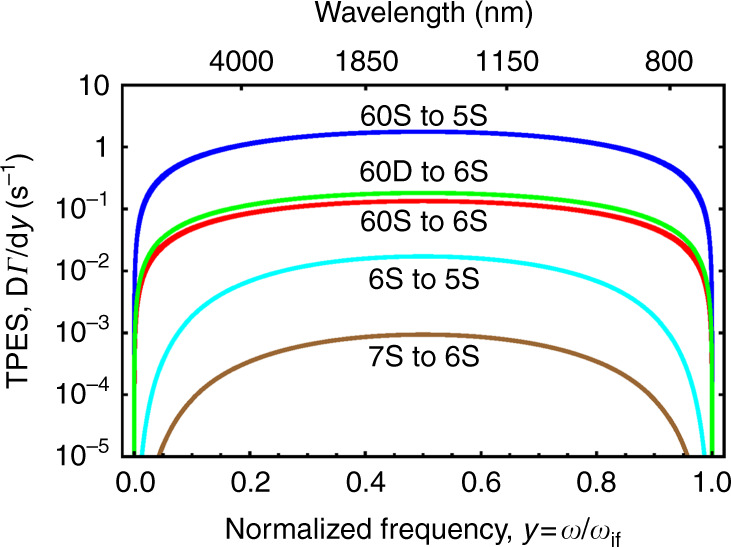


The original article has also been updated.
