# Supplementary material for: Creating heralded hyper-entangled photons using Rydberg atoms
Source: Light Sci Appl. 2021 May 12;10:100. doi: 10.1038/s41377-021-00537-2 (PMC8113235; doi:10.1038/s41377-021-00537-2)
Supplement: Supplementary file 1 — Supplementary information for Creating heralded hyper-entangled photons using Rydberg atoms [file 41377_2021_537_MOESM1_ESM.pdf]

Supplementary information for
Creating heralded hyper-entangled photons using Rydberg atoms

Sutapa Ghosh[1], Nicholas Rivera[2], Gadi Eisenstein[1] and Ido Kaminer[1]

[1]*Andrew and Erna Viterby Department of Electrical Engineering and Russell Berrie Nanotechnology Institute, Technion-Israel Institute of Technology, Haifa 32000, Israel*

[2]*Department of Physics, Massachusetts Institute of Technology, Cambridge, MA, United States*

I. TWO-PHOTON EMISSION (TPE) RATES CALCULATED USING MACROSCOPIC QED

In this section, we recall the QED formalism to calculate the two-photon spontaneous decay rate of an atom. We consider an atom initially in an excited state $|i\rangle$ that decays to the final state $|f\rangle$. The Hamiltonian representing the atom-light interaction is $H_{int} = \sum\limits_i \frac{e}{2m_e}(p_i \cdot A + A \cdot p_i) + \frac{e^2}{2m_e^2}A^2$. Here, we can neglect the A^2 term as its contribution for TPE rates is very small compared to a second order perturbation theory with the first term. The unitary operator for the atom-light interaction is given by:

$$U(t,t_0) = e^{-\frac{i}{\hbar}\int_0^t H'_{int}(t')dt'} = 1 - \frac{i}{\hbar}\int_0^t H'_{int}(t')dt' + \frac{1}{\hbar^2}\int_0^t H'_{int}(t')dt'\int_0^{t'} H'_{int}(t^{''})dt^{''} + \text{higher orders}$$

The first term in the above equation represents the zero order processes while the second term represents the first order processes which give the Fermi Golden rule for spontaneous emission rates. For the TPE rates, we consider the third term that represents the decay of atoms by two photons. The transition amplitude is written as:

$$\begin{aligned} S_{i\to f} &= \langle f|U(t,t_0)|i\rangle \\ &= -\frac{1}{\hbar^2}\langle f|\int_0^t H'_{int}(t')dt'\int_0^{t'} H'_{int}(t'')dt''|i\rangle \\ &= -\frac{1}{\hbar^2}\sum_m \langle f|\int_0^t H'_{int}(t')dt'|m\rangle\,\langle m|\int_0^{t'} H'_{int}(t'')dt''|i\rangle \end{aligned}$$

The state $|m\rangle$ represents the contribution of all the energy levels.

In the interaction picture, the Hamiltonian is $H'_{int}(t) = e^{iH_0t}H_{int}(t)e^{-iH_0t}$ where $H_0 = H_{atom} + H_{field}$.

$$S_{i\to f} = -\frac{1}{\hbar^2}\sum_m \langle f|H^{(2)}_{int}|m\rangle\,\langle m|H^{(1)}_{int}|i\rangle \int_0^t e^{i(\omega_2-\omega_{mf})t'}dt' \int_0^{t'} e^{i(\omega_1-\omega_{im})t''}dt''$$

ω_1 and ω_2 are the energies of the first and second photons and ω_{if} is the energy difference between the transition level $|i\rangle$ and $|f\rangle$. The two photon transition probability is given as:

$$P_{i\to f} = |S_{i\to f}|^2 = \frac{2\pi t}{\hbar^4}\Big|\sum_m \frac{\langle f|H^{(2)}_{int}|m\rangle\,\langle m|H^{(1)}_{int}|i\rangle}{(\omega_1-\omega_{im})} + \frac{\langle f|H^{(1)}_{int}|m\rangle\,\langle m|H^{(2)}_{int}|i\rangle}{(\omega_2-\omega_{im})}\Big|^2 \delta(\omega_1+\omega_2-\omega_{if})$$

The TPE rate in terms of density of states $\rho(\omega)$ is given by:

$$\frac{d\Gamma}{d\omega_1} = \lim_{t\to\infty}\frac{1}{t}P_{i\to f} = \frac{2\pi}{\hbar^4}\Big|\sum_m \frac{\langle f|H^{(2)}_{int}|m\rangle\,\langle m|H^{(1)}_{int}|i\rangle}{(\omega_1-\omega_{im})} + \frac{\langle f|H^{(1)}_{int}|m\rangle\,\langle m|H^{(2)}_{int}|i\rangle}{(\omega_{if}-\omega_1-\omega_{im})}\Big|^2 \rho(\omega_1)\rho(\omega_{if}-\omega_1) \tag{S1}$$

II. TPE RATES OF AN ATOM IN FREE SPACE

In this section, we follow a known derivation for the TPE rates of an atom in free space. In Supplementary section V, we extend this derivation for an atom in a cavity. The density of vacuum modes (for each polarization) is given by: $\rho(\omega) = \frac{\omega^2 d\omega d\Omega}{(2\pi c)^3/V}$, where $d\Omega$ is the solid angle covered by the emitted photons. From Eqn. S1, the TPE rate under the dipole approximation is given as:

$$\Gamma = \frac{2\pi}{\hbar^4} \int \Big| \sum_m \frac{\langle f|H_{int}^{(2)}|m\rangle \langle m|H_{int}^{(1)}|i\rangle}{(\omega_1 - \omega_{im})} + \frac{\langle f|H_{int}^{(1)}|m\rangle \langle m|H_{int}^{(2)}|i\rangle}{(\omega_2 - \omega_{im})} \Big|^2 \delta(\omega_1 + \omega_2 - \omega_{if}) \\ \frac{k_1^2 k_2^2 dk_1 dk_2 sin\theta_1 sin\theta_2 d\theta_1 d\theta_2 d\phi_1 d\phi_2}{(2\pi)^3/V(2\pi)^3/V}$$

$$\Gamma = \frac{1}{18\pi^3 c\hbar^2\epsilon_0^2} \int_{k_1=0}^{k_{if}} k_1^3 (k_{if} - k_1)^3 dk_1 \Big| \sum_m d_{fm} d_{mi} \Big(\frac{1}{k_1 - k_{im}} + \frac{1}{k_{fi} - k_1 - k_{im}} \Big) \Big|^2$$

where $k = \omega/c$ and d_{fm} is the dipole matrix for transition from state $|m\rangle$ to $|f\rangle$. For simplicity, we define $y = k_1/k_{if}$ and $y_{im} = \frac{E_i - E_m}{E_i - E_f}$. The TPE rate is averaged over all the directions and polarizations.

$$\begin{aligned} \Gamma &= \frac{e^4 a_0^4 k_{if}^5}{18\pi^3 c\hbar^2\epsilon_0^2} \int_{y=0}^{1} y^3 (1-y)^3 dy \Big| \sum_m d_{fm} d_{mi} \Big(\frac{1}{y - y_{im}} + \frac{1}{1 - y - y_{im}} \Big) \Big|^2 \\ &= \frac{e^4 a_0^4 k_0^5}{18\pi^3 c\hbar^2\epsilon_0^2} \Big(\frac{k_{if}}{k_0} \Big)^5 \int_{y=0}^{1} y^3 (1-y)^3 dy \Big| \sum_m d_{fm} d_{mi} \Big(\frac{1}{y - y_{im}} + \frac{1}{1 - y - y_{im}} \Big) \Big|^2 \end{aligned}$$

Here, $k_0 = \frac{3Z^2\alpha}{8a_0}$ is the energy difference of the hydrogen atom between states 1S and 2S, and a_0 is the Bohr radius. The coefficient of the TPE rate can be rewritten in terms of constants as:

$$C = \frac{e^4 a_0^4 k_0^5}{18\pi^3 c\hbar^2\epsilon_0^2} = \frac{3^2 Z^{10}}{2^{10}} R_H \alpha^6 c$$

where $R_H = \frac{m_e e^4}{8\epsilon_0^2 h^3 c}$ is the Rydberg constant, $\alpha = \frac{e^2}{4\pi\epsilon_0 \hbar c} = \frac{\hbar}{m_e c a_0}$ is the fine-structure constant and Z is the atomic number. The total TPE rate is given by:

$$\Gamma = \frac{3^2 Z^{10}}{2^{11}} R_H \alpha^6 c \Big(\frac{k_{fi}}{k_0} \Big)^5 \int_{y=0}^{1} y^3 (1-y)^3 dy \Big| \sum_m d_{fm} d_{mi} \Big(\frac{1}{y - y_{im}} + \frac{1}{1 - y - y_{im}} \Big) \Big|^2 \tag{S2}$$

III. RUBIDIUM RYDBERG BOUND STATE WAVE FUNCTIONS

This section calculates the dipole matrix for different transitions by solving numerically the wave functions for the rubidium atom. The radial part of the wave function is calculated from the Schrodinger equation as:

$$\frac{\partial^2 R_{nl}}{\partial r^2} + \frac{2}{r}\frac{\partial R_{nl}}{\partial r} + \left[2E_n + 2V(r) - \frac{l(l+1)}{r^2}\right]R_{nl} = 0 \tag{S3}$$

For the hydrogen atom, the $V(r)$ represents the Coulomb potential $(-1/r)$. For the rubidium atom, one has to add the effect due to the non-negligible penetration of the outer electron into the electronic cloud of the core. Therefore, $V(r)$ is modeled as:

$$V(r) = -\frac{Z_l(r)}{r} - \frac{\alpha_c}{2r^4}(1 - e^{-(r/r_c)^6}) + V_{SO}$$

where $V_{SO}(r) = \alpha\frac{L.S}{(2r)^3}$ is the relativistic spin-orbit interaction. α_c is the core polarizability, r_c is a cutoff radius introduced to avoid divergence at short range and $Z_l(r)$ is the effective charge given by $Z_r(l) = 1 + (Z-1)e^{-a_1 r} - r(a_3 + a_4 r)e^{-a_2 r}$ with l-dependent parameters a_l which are obtained by fitting the model potential to measured energy levels for each element [1]. The modified energy levels are given by:

$$E_{n^*} = -\frac{R_\infty}{1+\frac{m_e}{M}}\frac{1}{2(n-\delta_{nlj})} = -\frac{R_\infty}{1+\frac{m_e}{M}}\frac{1}{2n^*}$$

where $n^* = n - \delta_{nlj}$ is the principle quantum number corrected by the quantum defect given by,

$$\delta_{nlj} = \delta_{lj,0} + \frac{\delta_{lj,2}}{(n-\delta_{lj,0})^2} + \frac{\delta_{lj,4}}{(n-\delta_{lj,0})^4} + \frac{\delta_{lj,6}}{(n-\delta_{lj,0})^6} + ...$$

Eqn. S3 is solved numerically by using a Numerov method. The integral in r starts from the outer radius at a distance $r = 2n(n+15)a_0$ from the atomic core, where the wave function exponentially decays to zero. The integral is carried out inwards towards $r = 0$. An initial solution is assumed for the radial wave function which converges to the physical solution iteratively. Due to the nonzero quantum defect parameters δ, the wave function diverges at small r. Since the contribution at small r is negligible, the integral is stopped when it starts to diverge. In order to solve the wave function and to calculate the dipole matrix we have used various Python libraries developed by [1].

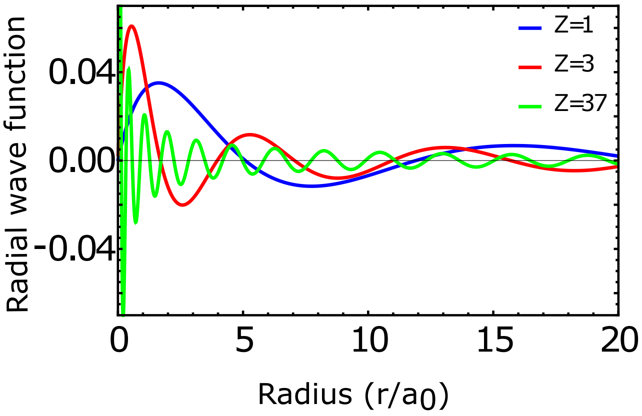

Fig. S1. **Continuum wave functions for hydrogenic atoms**. The plot is for continuum states with $Z = 1$ (blue), 3 (red) and 37 (green) for momentum $p = 0.001$. As Z increases, the wave function oscillates fast, which effectively reduces its contribution for the TPE rates.

IV. THE EFFECT OF THE CONTINUUM STATES

The dipole matrix element contain the contributions of the bound states and continuum states. In case of the hydrogen atom, it has been shown that the contributions of continuum states are quite small but not negligible [2]. For calculating the continuum states we modelled the potential as $V(r) = -\frac{Z}{r}$ (this approximation is considered accurate for the continuum states; our model for the bound state does not rely on this approximation). The analytical expression for the continuum state for hydrogenic atom is [3]:

$$R_{pl}^{cont} = \frac{C_{pl}}{(2l+1)!}(2pr)^l e^{-ipr} F(iZ/p + l + 1, 2l + 2, 2ipr)$$

$$C_{pl} = 2pe^{\pi Z/2p}|\Gamma(l + 1 + iZ/p)|,$$

where F represents a first order hypergeometric function and $p = \sqrt{2E}$ represents the continuous momentum states in atomic units. For TPE rates, we calculate the overlap wave function of the initial and final bound states with the continuum states for the p-state $l = 1$. Through this calculation, we found that for rubidium atoms, the contributions of the continuum states are negligible. The reason is that the wave functions of the continuum states for rubidium oscillate much faster than for states with lower atomic number Z as shown in Fig. S1. Consequently, their contribution decreases with increasing Z as summarized in Table S1. We found that only $Z = 1$ contributes for TPE rates while the higher Z has no effect on TPE rates. For an ideal calculation, $V(r)$ should be modified for the rubidium atom. But we have seen that for higher Z, the effect of the continuum states is negligible. Thus, this should not affect the TPE rates.

Transition	TPE wo continuum (s^{-1})	Z	continuum states (s^{-1})
$60S_{1/2} \to 6S_{1/2}$	0.0447015	1	0.0554188
		3	0.0447019
		11	0.0447018
		37	0.0447018

Table S1. **Contribution of the continuum states to the TPE rates in the rubidium atom**. The TPE rates are calculated by adding the contributions of continuum states for different Z for comparison. With the increase of Z, these contributions becomes negligible.

V. TPE OF ATOMS INSIDE AN OPTICAL CAVITY

This section derives the TPE rates for atoms inside an optical cavity with length L and decay rate κ. The density of optical modes around the atom (for each polarization) is modified by the cavity and is written as: $\rho_{cav} = \frac{1}{\pi}\sum_n \frac{\omega^2\kappa}{(\omega_{cn}^2-\omega^2)^2+\omega^2\kappa^2} = \frac{1}{\pi}\sum_n \frac{1/\kappa}{1+\left(\frac{\omega_{cn}^2-\omega^2}{\omega\kappa}\right)^2}$, where ω_{cn} is the nth cavity mode frequency. The TPE rate is thus:

$$\Gamma = \frac{2\pi}{\hbar^4}\int d\omega_1 d\omega_2 \Big|\sum_m \frac{\langle f|H_{int}^{(2)}|m\rangle\,\langle m|H_{int}^{(1)}|i\rangle}{\omega_1-\omega_{im}} + \frac{\langle f|H_{int}^{(1)}|m\rangle\,\langle m|H_{int}^{(2)}|i\rangle}{\omega_2-\omega_{im}}\Big|^2$$
$$\sum_{n1,n2} \frac{\delta(\omega_1+\omega_2-\omega_{if})}{(\pi\kappa)^2\Big[1+\left(\frac{\omega_{cn1}^2-\omega_1^2}{\omega_1\kappa}\right)^2\Big]\Big[1+\left(\frac{\omega_{cn2}^2-\omega_2^2}{\omega_2\kappa}\right)^2\Big]}$$

Let us define $y=\omega_1/\omega_{if}$, $y_{im}=\omega_{im}/\omega_{if}$, $y_{cn}=\omega_{cn}/\omega_{if}$ and $\Delta y = \kappa/\omega_{if}$.

$$\Gamma = \frac{k_0^5 e^4 a_0^4}{2\pi\hbar^2 c\epsilon_0^2}\Big(\frac{k_{if}}{k_0}\Big)^5\Big(\frac{1}{Vk_{if}^3}\Big)^2\int_0^1 y(1-y)dy\sum_{n1,n2}\Big(\frac{\omega_{cn1}}{\kappa}\Big)\Big(\frac{\omega_{cn2}}{\kappa}\Big)\Big(\frac{\omega_{if}}{\omega_{cn1}}\Big)\Big(\frac{\omega_{if}}{\omega_{cn2}}\Big)$$
$$\frac{1}{\Big[1+\left(\frac{y_{cn1}^2-y^2}{y\Delta y}\right)^2\Big]\Big[1+\left(\frac{y_{cn2}^2-(1-y)^2}{(1-y)\Delta y}\right)^2\Big]}\Big|\sum_m d_{fm}d_{mi}\Big(\frac{1}{y-y_{im}}+\frac{1}{1-y-y_{im}}\Big)\Big|^2$$

The dipole moment matrix is in units of ea_0. The coefficient of TPE can be rewritten in terms of constants as:

$$C = \frac{k_0^5 e^4 a_0^4}{2\pi\hbar^2 c\epsilon_0^2} = \frac{9\pi^2 9 R_H\alpha^6 c}{2^{10}} = \frac{3^4\pi^2 R_H\alpha^6 c}{2^{10}}$$

The TPE rate can be finally written as:

$$\Gamma = \frac{3^4\pi^2 R_H\alpha^6 c}{2^{11}}\Big(\frac{k_{if}}{k_0}\Big)^5\Big(\frac{1}{Vk_{if}^3}\Big)^2\int_0^1 y(1-y)dy\sum_{n1,n2}\Big(\frac{\omega_{cn1}}{\kappa}\Big)\Big(\frac{\omega_{cn2}}{\kappa}\Big)\Big(\frac{\omega_{if}}{\omega_{cn1}}\Big)\Big(\frac{\omega_{if}}{\omega_{cn2}}\Big)$$
$$\frac{1}{\Big[1+\left(\frac{y_{cn1}^2-y^2}{y\Delta y}\right)^2\Big]\Big[1+\left(\frac{y_{cn2}^2-(1-y)^2}{(1-y)\Delta y}\right)^2\Big]}\Big|\sum_m d_{fm}d_{mi}\Big(\frac{1}{y-y_{im}}+\frac{1}{1-y-y_{im}}\Big)\Big|^2$$

The photons emitted from the atom resonant with the cavity are emitted along the cavity axis. Also, the polarization of the emitted photons depends on the quantization axis which can be defined by applying an external magnetic field to the atoms. To add an additional degree of freedom to the entangled state, we propose to choose the quantization axis perpendicular to the cavity axis, so the cavity only supports σ^+ and σ^- polarizations.

VI. HONG-OU-MANDEL INTERFERENCE FOR QUDIT STATES

In this section, we derive the analytical form for Hong-Ou Mandel interference with N-dimensional energy entangled states. The entanglement between different energy modes gives rise to interference which depends on the dimension of the entangled states. We present a concrete example for the required calculation with $N = 4$.

$$|\psi\rangle_f = \frac{1}{2}(a^\dagger_{c,1}a^\dagger_{d,-1} + a^\dagger_{c,-1}a^\dagger_{d,1} + a^\dagger_{c,2}a^\dagger_{d,-2} + a^\dagger_{c,-2}a^\dagger_{d,2})\,|0,0\rangle\,.$$

The total electric field at each of the input ports of the beam splitter BS are denoted by c and d. In each port the field is a superposition of all the energy modes with frequency ω

$$E^+_k = \frac{1}{\sqrt{2}}(a_{k,1}e^{-i\omega_1 t} + a_{k,2}e^{-i\omega_2 t} + a_{k,-1}e^{-i\omega_{-1} t} + a_{k,-2}e^{-i\omega_{-2} t})$$

The observation of quantum interference in two-photon coincidence measurements as a function of delay between the two photon paths confirms the entanglement between the energy modes. Thus, the time-resolved two-photon coincidence is detected when both detectors clicks; it is defined as:

$$\begin{aligned}
P_2(\tau) &= \langle E^-_d(t)E^-_c(t+\tau)E^+_c(t+\tau)E^+_d(t)\rangle \\
&= \frac{1}{4}\Big[1 + \frac{2}{4}(\cos(\omega_1-\omega_{-1})\tau + \cos(\omega_2-\omega_{-2})\tau + \cos(\omega_1-\omega_{-2})\tau + \cos(\omega_2-\omega_{-1})\tau \\
&\quad + \cos(\omega_1-\omega_2)\tau + \cos(\omega_2-\omega_{-2})\tau)\Big]
\end{aligned}$$

For N entangled pairs, the coincidence probability is generalized as

$$P_2(\tau) = \frac{1}{N}\Big[1 + \frac{2}{N}\sum_{\substack{i=1\\ i\neq j}}^{N/2}\sum_{j=1}^{N/2}\cos(\omega_i-\omega_j)\tau\Big]$$

For an atom inside a cavity, the frequency modes of the N-dimensional photon comb are separated by the cavity's free spectral range ν_{FSR}. Therefore, the coincidence probability for $N = 10$ comb lines is

$$\begin{aligned}
P_2(\tau) = \frac{1}{10}\Big[1 + \frac{2}{10}(&9\cos(2\pi\nu_{\mathrm{FSR}}\tau) + 8\cos(4\pi\nu_{\mathrm{FSR}}\tau) + 7\cos(6\pi\nu_{\mathrm{FSR}}\tau) + 6\cos(8\pi\nu_{\mathrm{FSR}}\tau) + 5\cos(10\pi\nu_{\mathrm{FSR}}\tau) \\
&+ 4\cos(12\pi\nu_{\mathrm{FSR}}\tau) + 3\cos(14\pi\nu_{\mathrm{FSR}}\tau) + 2\cos(16\pi\nu_{\mathrm{FSR}}\tau) + \cos(18\pi\nu_{\mathrm{FSR}}\tau))\Big]
\end{aligned}$$

This equation is used in Fig. 4 to represent the coincidence probability for 10-dimensional entangled photons.

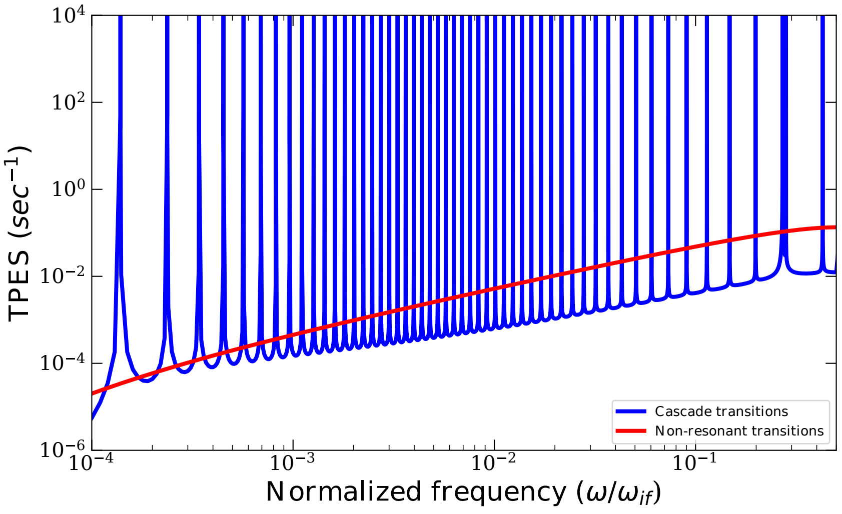

Fig. S2. **Effect of the resonant cascade transitions for $60S_{1/2} \rightarrow 6S_{1/2}$, before they are inhibited by the cavity.** The vertical axis is the rate of TPE per unit normalized frequency. The contribution for the cascade transitions (blue) from the energy levels lying between the initial and final states, while the non-resonant contributions are from the energy levels higher than the initial state (red). The resonance peaks for the cascade transitions are suppressed by the cavity. The background added to the TPE rates is 10 times smaller than the main TPE.

VII. EFFECT OF RESONANT CASCADE PROCESSES AND CAVITY INHIBITED TRANSITIONS

The TPE processes can be mediated either by non-resonant processes where the atom decays through a higher energy level, or by resonant cascade processes where the atom decays through an energy state lower than the initial state. Usually, the non-resonant processes are very slow (in free space, without a cavity that enhances them) compared to the resonant ones. The resonant cascade processes involve transitions from real levels, which means that the photons are not emitted instantaneously. Instead, these processes generate energy-time correlations that depend on the lifetime of the intermediate states [4, 5]. In our protocol, we suppress the contribution of the resonant cascade processes through an optical cavity where the cavity length is tuned ($L = 98.417\ \mu m$) such that most of these resonant fast processes are suppressed. Table S2 summarizes the transitions from $60S_{1/2}$. Thus nearly all TPE processes are due to the virtual transitions which lead to strongly entangled pairs. There are still some higher order cascade processes that are not suppressed, however their rates are lower than the TPE rates that produce our entangled photon pairs. Future designs can consider cavities with a broader spectral range that can be used to inhibit additional transitions. In Fig. S2, it is shown that the background of the cascade two-photon emission spectrum is 10 times smaller than the non-resonant one. Thus, the main contribution to the TPE rates comes from the non-resonant processes.

Transitions	Wavelength (nm)	Transition rate, Γ_{free} (s^{-1})	Cavity inhibition ratio, $\Gamma_{cav}/\Gamma_{free}$
$60S_{1/2} \rightarrow 5P_{3/2}$	479.8389	1338.13	7.74 $\times 10^{-10}$
$60S_{1/2} \rightarrow 5P_{1/2}$	474.4301	702.19	1.98 $\times 10^{-9}$
$60S_{1/2} \rightarrow 6P_{3/2}$	1013.7585	516.83	5.49 $\times 10^{-9}$
$60S_{1/2} \rightarrow 6P_{1/2}$	1005.8549	275.00	1.37 $\times 10^{-9}$
$60S_{1/2} \rightarrow 7P_{3/2}$	1728.0815	271.07	4.08 $\times 10^{-8}$
$60S_{1/2} \rightarrow 7P_{1/2}$	1717.6649	145.46	2.39 $\times 10^{-9}$
$60S_{1/2} \rightarrow 8P_{3/2}$	2629.6476	163.77	4.16 $\times 10^{-8}$
$60S_{1/2} \rightarrow 8P_{1/2}$	2616.6793	88.411	1.87 $\times 10^{-8}$
$6S_{1/2} \rightarrow 5P_{3/2}$	1366.8737	1.43 $\times 10^{7}$	2.35 $\times 10^{5}$

Table S2. **Cavity inhibited one photon transitions from $60S_{1/2}$.** We use a cavity with finesse 1.2×10^6 and length 98.417 μm such that the first order processes faster than TPE processes has been inhibited while the heralding transition $6S_{1/2} \rightarrow 5P_{3/2}$ is enhanced.

[1] N. Šibalić, J.D. Pritchard, C.S. Adams, and K.J. Weatherill, "Arc: An open-source library for calculating properties of alkali rydberg atoms," Computer Physics Communications **220**, 319 – 331 (2017).

[2] J. Chluba and R. A. Sunyaev, "Two-photon transitions in hydrogen and cosmological recombination," Astronomy and Astrophysics **480**, 629–645 (2008).

[3] L. D. Landau and E. M. Lifshitz, *Quantum Mechanics - Non relativistic theory, Ed. 3* (Pergamon, London, 1977).

[4] M. Scully and M. Zubairy, *Quantum Optics* (Cambridge: Cambridge University Press, 1997).

[5] Daniel Boyanovsky and Louis Lello, "Time evolution of cascade decay," New J. Phys. **16**, 063050 (2014).
